# Effect of bone density on the survival of 407 sandblasted and acid-etched dental implants: A retrospective multicenter study

**DOI:** 10.34172/joddd.2023.39248

**Published:** 2023-07-17

**Authors:** Jean-Marc Foletti, Manon Sterba, Pascal Maurice, Jean-Christophe Dibatista, Raphael de Gea, Laurine Birault

**Affiliations:** ^1^Aix Marseille University, University Gustave Eiffel, LBA, Marseille, France; ^2^Department of Oral and Maxillofacial Surgery, APHM, Conception University Hospital, Marseille, France; ^3^Glad Medical SAS, Salon-de-Provence, France; ^4^Private Dental Practice 53 Route d’Uzès, 30000 Nîmes, France; ^5^Private Dental Practice, Rond-Point du Général Diego Brosset, 83580 Gassin, France; ^6^Private Dental Practice, 18 Chemin de Saint-Marc, 83150 Bandol, France; ^7^Private Dental Practice, 455 Promenade des Anglais, L’Arénas 06200 Nice, France

**Keywords:** Bone density, Clinical study, Dental implants, Dentistry, Implant survival rate, Implantology

## Abstract

**Background.:**

This study evaluated the success and survival rate of sandblasted and acid-etched dental implants according to the patient’s bone quality.

**Methods.:**

A multicenter retrospective study was conducted in five clinical centers between 2016 and March 2019. A total of 407 implants (KONTACT^TM^ S, Biotech Dental, France) placed in 229 patients (61.5±12.9 years old) were included. Bone quality, classified as types D1 to D4 (Misch classification), maximal insertion torque, and bone loss were measured. The implant survival rate was evaluated after one year for the overall cohort and for each bone quality. The overall survival rate after four years was also estimated with a Kaplan-Meier analysis.

**Results.:**

After one year (12.8±9.6 months), eight implants were lost out of 407, representing an overall survival rate of 98%. It ranged from 100% for D1 to 89.7% for D4 (n=39), with significantly higher survival rates for D2 (n=93) and D3 (n=165) (98.9% and 98.2%, respectively) compared to D4. According to the Kaplan-Meier analysis, an overall survival rate of 96.5% was estimated after four years. An average maximal insertion torque of 45±12.6 N.cm and bone loss of 0.2±1.2 mm were measured.

**Conclusion.:**

The high overall survival rate (98%), the average maximal insertion torque (45 N.cm), and the low marginal bone loss indicated good clinical results with acid-etched implants. Despite the relatively high survival rate for each bone quality, the significantly lower results in the D4 group highlight the expected benefits of bone quality-based implants and surgical protocols.

## Introduction

 Dental implants have been worldwide solutions for tooth loss replacement for decades. Total osseointegration is mandatory for long-term implant success. Among the parameters favoring osseointegration, the patient’s bone characteristics, especially bone density, are significantly correlated to both primary stability and osseointegration success in the literature.^1-4^

 The final success of implant rehabilitation is multifactorial and still possible even with low bone density. Implants’ intrinsic factors can improve primary stability, biocompatibility, and osseointegration in all bone types. Surface modification treatments, in particular, have demonstrated their crucial importance. Subtractive techniques create surface roughness which favors immediate bone anchoring of the implant and promotes quality cell growth and adhesion to improve the bone-to-implant contact (BIC).^5^ Both primary stability and osseointegration are optimized compared to an untreated implant.^6,7^ The most common subtractive technique associates sandblasting (several substances were used, i.e., aluminum or calcium phosphate particles) and acid-etching. Microscopic refinements are currently compared in the literature.

 The beneficial effects of sandblasting on osseointegration are supported by clinical data, with excellent long-term survival rates in the literature. Blasting affords titanium roughness which favors primary mechanical stability; sandblast-modified surfaces increase the BIC compared to old machined and modified surfaces. Goiato et al^3^ reported a higher survival rate in type IV bone for sandblasted, roughened implants than for modified implant surfaces. Acid-etching (AE) methods lead to a uniform implant surface, with micro-pits of approximately 1‒3 µm in width; this technique has shown potential in improving bone-to-implant fixation due to an increase in bioactivity on the implant surface.^8^ The superiority of AE surface on the machined surface is confirmed in the literature regarding both primary stability and survival^9^ with a moderate influence of bone density on implant success.^10^

 As the blasting provides roughness optimal for mechanical fixation, additional etching further modifies surface topography and chemistry. Acid etching theoretically eliminates the surface pollution resulting from blasting and adds further surface micro- and nano-irregularities on the implant surface with positive effects on the activation of blood platelets and cell migration.^11,12^ A combination of sandblasting and acid etching enhances implants’ hydrophilicity, favoring biocompatibility in the early bone formation stage and shortening the osseointegration process.^13^

 Wang et al^9^ confirmed how multi-scale surface modification techniques could shorten the bone ingrowth phase. They, however, highlighted the need to confirm these proven benefits of modified surfaces through clinical studies. Moreover, as several research groups are studying the effects of micro-scale optimizations of surface treatments on titanium implants’ osseointegration, only a few have focused their attention on the benefits of such surface treatments in various densities of bone. Despite the expected ability of modified surfaces to enhance osseointegration, increasing the BIC, even in compromised and at-risk conditions, is supported by clinical data and histological evidence. However, there are concerns about the possible influence of surface characteristics on long-term peri-implant tissue health.^11,14^

 Here, we evaluated the success and survival rate of sandblasted and acid-etched dental implants according to the patient bone quality with a multicenter retrospective study.

## Methods

 This retrospective multicenter clinical study was carried out in full compliance with the Declaration of Helsinki of the World Medical Association, with the ISO 14155:2020, the Regulation EU 2017/745, Law #2016-41, Law #2019-774, and registered on the HDH & INDS platform. This study included patients treated for a single or multiple implant-supported rehabilitation with sandblastedand acid-etched* dental implants *(KONTACT^TM^ S, Biotech Dental, France) in five private dental centers between 2016 and March 2019 by six experienced surgeons. The data collection ended in 2021, ensuring a minimum of one-year follow-up for each patient. Implants included in this study were sandblasted and acid-etched implants in titanium grade 4 (T60 4B) with a diameter and length of 3.0 mm and 5.4 mm and 6 mm and 16 mm, respectively.

 Patients eligible for inclusion in this study were partially edentulous patients ( > 18 years old) needing implant treatment in the mandible or maxillary and presenting enough bone at the implant site to support it (with or without bone grafts). The exclusion criteria were poor oral hygiene at the time of surgery, general health conditions preventing surgical treatment, metabolic disorders or disorders affecting implant healing, bone, gingival and/or periodontal infections, inflammations, or diseases, immunosuppressive disorders or treatment, use of interfering medication (i.e., steroid therapy), and abuse of alcohol and/or drugs, and titanium allergy. Given the manufacturer’s instructions, cases of misuse, i.e., use of implants for patients with parafunction (such as bruxism) or smokers ( ≥ 10 cigarettes per day), were excluded from the study.

 The implant type (diameter, length), location, associated implant site characteristics, and loading protocol (immediate or delayed implant loading) were recorded for each implant and patient. Implant sites were divided into immediate post-extraction implant placement and previous edentulous with or without the need for a bone graft.

 For each implant, the bone quality at the implant site was classified into four types according to the classification of Misch.^15^ To ensure uniformity of the results, all the involved clinicians received the same instructions for classification.

 The survival rate, the mean maximal torque, and the mean marginal bone loss were measured to assess implant performance and security.

 The implant survival was considered in situ loaded implants at the evaluation time.^16^ Implants are considered failed if they need to be removed and replaced. Cumulative implant survival was evaluated at 12, 18, 24, and 48 months with a Kaplan-Meier analysis performed with IBM SPSS Version 27.0 (Armonk, NY: IBM Corp). The last implant follow-up (50 months) was used as the time for censoring for Kaplan-Meier analysis.

 The primary stability was evaluated as the maximal insertion torque at the time of implantation. A minimal insertion torque of 15 N.cm was considered in this study to establish sufficient primary stability. Moreover, the marginal bone loss was measured from the radiographs during the follow-up. Descriptive statistics (means and standard deviations) were performed with Microsoft Excel (Version 2104).

 To investigate the effect of bone quality, the survival rate was evaluated for each bone type and compared. The effect of implant site characteristics, implant location and type, and loading protocols were also evaluated. Statistical effects were tested using Fisher’s exact test performed with BiostaTGV (http://biostatgv.sentiweb.fr/). The level of significance was set at *P* = 0.05.

## Results

**Figure 1:**
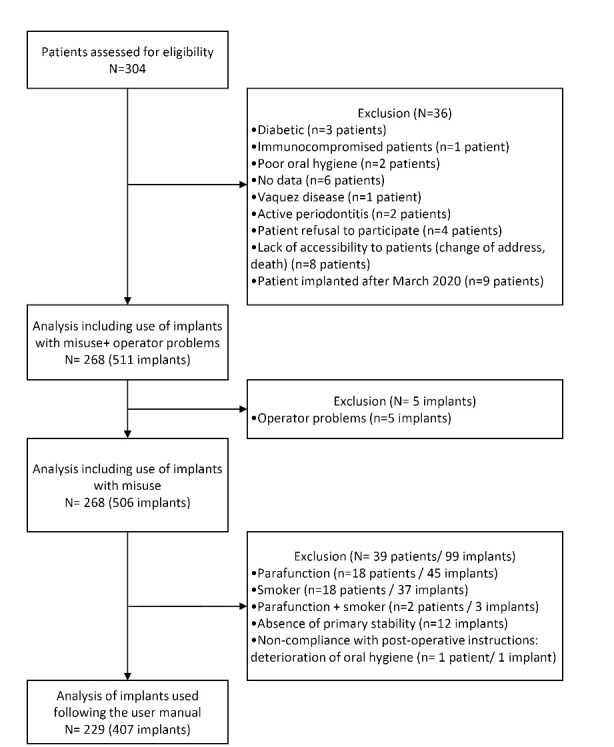
Flow chart of data selection

 A total of 407 implants (KONTACT^TM^ S, Biotech Dental, France) placed in 229 patients (mean age at the time of surgery: 61.5 ± 12.9 years old; 197 males and 210 females) were included in this study. A descriptive analysis regarding the implant selection is presented in Figure 1.

 Almost equivalent numbers of implants were placed in the maxilla and mandible (Table 1). The most common implant diameters used were 3.6 mm and 4.2 mm, representing 83% of placed implants. In terms of length, 79% of placed implants had a length of 10 mm or 12 mm. In terms of implant sites, 62 (15 %) were immediate post-extraction implant placement, and 339 implants (83%) were placed in previously edentulous sites. Bone graft was used for 156 implantations (38%). Finally, delayed loading was used for 332 implants (82%), and 75 (18%) were loaded immediately. Among the 407 implants, 26 (6.4%), 93 (22.9%), 165 (40.5%), and 39 (9.6%) were inserted in bone types D1, D2, D3, and D4, respectively. Bone density was not available for 84 implants. Detailed results are presented in Table 1.

**Table 1:** Descriptive analysis of the implant cohort (implant type, implant site characteristics, and implantation type)

**Implant localization**	
Mandible	206 (51%)
Maxilla	201 (49%)
**Implant diameter (mm)**	
3	12 (3%)
3.6	151 (37%)
4.2	189 (46%)
4.8	44 (11%)
5.4	11 (3%)
**Implant length (mm)**	
6	6 (1%)
8	53 (13%)
10	166 (41%)
12	154 (38%)
14	24 (6%)
16	4 (1%)
**Type of implantation**	
Immediate post-extraction	62 (15%)
Recent tooth loss ( < 6 months)	102 (25%)
Past tooth loss ( > 6 months)	237 (58%)
Anodontia	2 (0.5%)
UA	4 (1%)
**Bone grafting**	
Yes	156 (38%)
No	251 (62%)
**Surgical protocol**	
Immediate loading	75 (18%)
One-time deferred loading	237 (58%)
Two times	95 (23%)
Bone type	
D1	26 (6%)
D2	93 (23%)
D3	165 (41%)
D4	39 (10%)
UA	84 (21%)

###  Implant performance and security

 Among the 407 implants, eight implants were lost, representing a survival rate of 98% after one year (12.8 ± 9.6 months). According to the Kaplan-Meier analysis, a global survival rate of 96.5% was estimated after four years.

 The overall implant stability at implant insertion was 45 ± 12.6 N.cm (n = 229 implants). The implant stability was 50.7 ± 11.4 N.cm (n = 22 implants), 48.2 ± 12.3 N.cm (n = 84 implants), 40.8 ± 12.6 N.cm (n = 42 implants), and 45 ± 35.3 N.cm (n = 2 implants) for D1, D2, D3, and D4 respectively. The average bone loss after one year was 1.2 ± 0.2 mm (n = 251 implants, with eight for D1, 62 for D2, 144 for D3, and 37 for D4).

 According to the statistical analysis, the survival rate was significantly higher in D2 (98.9%) and D3 (98.2%) bone types compared to D4 (89.7%) (*P* < 0.05). The values are presented in Table 2.

**Table 2:** Survival rate analysis according to bone type

**Bone type**	**Follow-up time (day)**	**Survival rate (%)**
D1 (n = 30)	452	100%
D2 (n = 107)	497	98,9%^a^
D3 (n = 211)	418	98,2%^b^
D4 (n = 39)	341	89,7%^a,b^
Total (n = 407)	384	98%

^a^Indicates a significant difference (*P* < 0.05). ^b^ Indicates a significant difference (*P* < 0.05).

 In addition to the bone quality, the need for bone grafting and the implant location (type of maxilla) significantly affected the survival rate. In contrast, the patient’s gender and age, the center where the patient was treated, history of periodontal disease, implant size (length and diameter) and number, type of implantation, and surgical protocol did not demonstrate a significant effect (Table 3).

**Table 3:** Statistical analysis of the effect of patient, implant, and protocol characteristics on the implant survival rate

**Variable**	**Category**	**Number of implants (N=407 implants)**	**Survival**	***P*** **value**^a^
Age (y)^b^	≤ 55	133	128	0.12
	> 55	274	271	
Gender	Male	197	191	0.16
	Female	210	208	
History of periodontal disease	Yes	87	85	1
	No	248	242	
	UA	72	0	
Number of implants placed^b^	≤ 2	258	254	0.47
	> 2	149	145	
Bone grafting	Yes	156	150	0.06
	No	251	249	
Surgical protocol	Immediate loading	75	73	0.25
	1 Time delayed loading	237	231	
	2 Times delayed loading	95	95	
Type of implantation	Immediate post-extraction	62	59	0.10
	Recent tooth loss	102	100	
	Older tooth loss	237	235	
	Anodontia	2	2	
	UA	4	3	
Localization	Mandible	206	205	0.04
	Maxilla	201	194	
Bone site	Anterior maxilla	64	62	0.16
	Anterior mandible	36	36	
	Posterior maxilla	137	132	
	Posterior mandible	170	169	
Length (mm)	6	6	6	0.6
	8	53	53	
	10	166	162	
	12	154	151	
	14	24	23	
	16	4	4	
Diameter (mm)	3	12	12	0.65
	3.6	151	149	
	4.2	189	183	
	4.8	44	44	
	5.4	11	11	
Center	1	173	167	0.49
	2	70	69	
	3	83	83	
	4	62	61	
	5	19	19	

^a^Fisher’s test ^b^ Categorization of Adler et al.^16^

## Discussion

 A multicenter retrospective study was designed to evaluate the success and survival rate of sandblasted and acid-etched dental implants according to the patient’s bone quality. The overall survival rate of our cohort of 407 implants was 98% after one year. The best survival rates were observed for D1, D2, and D3 groups, while the survival rate measured for D4 was slightly lower (89.7%).

 The overall implant stability at implant insertion was 45 N.cm, and the average bone loss was 0.2 mm. This combination of good primary stability and low bone loss corroborates our study’s overall high survival rate of implants. A slight decrease in primary stability for D3 and D4 compared to D1 and D2 was noticed. Recent literature data insists on the relationship between bone density and primary stability, with a growing interest in technics such as osseodensification,^17^ as it is quite admitted that a high-level bone density favors primary stability^4^; on the contrary, a low bone density may affect both primary stability and osseointegration success negatively.

 These findings corroborate the effect of bone quality on the survival rate and support the benefits of using bone-quality-based implants: Among other factors, microscale surface treatment can significantly improve the implant’s primary stability and osseointegration success.^3^ The progress in titanium surface treatments has enlarged clinical indications for dental implants to conditions formerly considered at risk of failure, such as patients with low bone density. The ability of SAE surface treatment to enhance osseointegration, increasing the BIC even in compromised and at-risk conditions, is nowadays supported by both clinical data and histological evidence.^4^

 Clinical and histological evidence supports the relationship between primary stability and bone density: In a 2015 review, Molly^2^ demonstrated that primary stability measurements showed significant correlations with different bone densities and implant outcomes. Radiologic-based methods and techniques are the most used because they are more elaborate in clinical practice. However, these methods may lack sensibility, except for very low-density bone. Also, the determination of bone density through radiography remains subjective and operator-dependent. On the other hand, no real alternative is available yet, as histomorphometric analysis of bone biopsies is difficult to implement in daily clinical practice.^2^

 A bone loss of up to 2 mm during the first year of loading should be considered early periimplantitis.^4^ The results of the present study regarding bone loss are inferior to the acceptable threshold admitted in classic implant success criteria ( < 1.5 mm of bone loss during the first year of loading and < 0.2 mm annually after the first year). This stability suggests a preventive role of the implants used in this study to prevent early peri-implantitis. We hypothesize this finding could be explained by the combination of the concept of platform switching associated with a seal provided by the Morse-cone-type connection, the global shape of the implant, and the surface treatment, as the relative impact of each factor is still debated in the literature.^18,19^ It has been demonstrated that variations in the prosthetic features result in different stress and strain values in the surrounding bone, with an even higher impact than the implant diameter.^20^ This point is of crucial importance regarding the primary stability and implant success in low-density bone (D4): according to Putra et al,^21^ D4 bone density may affect the accuracy of implant placement and therefore affect primary stability or increase bone loss and peri-implantitis risk. This may explain the results obtained in our series for D4 bone. Precise implant placement and appropriate prosthetic protocols are necessary for the optimal effectiveness of SAE implants in low-density bone.

 The multicenter recruitment increased the size of our sample and, thereby, the power of the study, but it could also constitute a risk of bias. As stated previously, bone density is determined based on a necessarily subjective and operator-dependent radiographic analysis. Likewise, the surgical technique of each operator can theoretically influence primary stability and implant survival rate. To minimize this limitation in our study, the protocol for radiographic analysis of bone density was standardized among the practitioners participating in the study by similar information about the Misch classification, and the same surgical drilling protocol, in conformity with the manufacturer’s recommendations, was performed by each practitioner.

 The results were obtained during an average one-year follow-up. Given that the implant was commercialized in 2016, a longer follow-up time should not lead to enough patients. To estimate the results for long-term follow-ups, A Kaplan-Meier analysis was performed. According to this analysis, a global survival rate of 96.5% was estimated after four years. This strategy of estimation has already demonstrated its reliability,^22^ including in the field of dental implantology.^23^

 The practitioner must consider the bone density of the treated site, identifying situations at risk of diminished primary stability (low bone density, grafted areas). Even with an adequate, rigorous surgical and prosthetic protocol, choosing the appropriate implant is one of the keys to improving long-term success. The results of our study enhance the multifactorial aspect of success in implantology. Some of these factors are related to the implant, considered both in its macrostructure (global design, composition, and dimensions) and on a microscopic scale (surface treatment, as SAE here); other factors are related to the patient, especially bone quality and density, not to mention systemic factors.

## Acknowledgments

 We especially thank BIOTECH DENTAL, which contributed to the study by providing the materials and equipment necessary for surgeries. We thank Dr. Michel Layet for contributing to data collection.

## Competing Interests

 The authors report no conflicts of interest.

## Ethical Approval

 This retrospective multicenter clinical study was conducted in accordance with the World Medical Association Declaration of Helsinki and the relevant parts of ISO 14155-2020.

## Funding

 This study was financially supported by GLAD MEDICAL SAS.
